# S1 Guideline on Infected Interdigital Intertrigo (also called Gram‐Negative Toe Web Infection)

**DOI:** 10.1111/ddg.70040x

**Published:** 2026-04-01

**Authors:** Christoph Zeyen, Dietrich Abeck, Karsten Becker, Joachim Dissemond, Birgit Kahle, Hans Peter Lorenzen, Bettina Löffler, Andreas Maier‐Hasselmann, Till Mittank‐Weidner, Alexander Nast, Cord Sunderkötter

**Affiliations:** ^1^ Department of Dermatology, Venereology and Allergology, Division of Evidence‐based Medicine (dEBM) Charité –Universitätsmedizin Berlin Corporate Member of Freie Universität Berlin Humboldt‐Universität zu Berlin Berlin Germany; ^2^ Dermatology Center Nymphenburg Munich Germany; ^3^ Friedrich Loeffler Institute of Medical Microbiology University Medicine Greifswald Greifswald Germany; ^4^ Department of Dermatology, Venereology and Allergology University Medical Center Essen Essen Germany; ^5^ Department of Dermatology, Venereology and Allergology University Medical Center Schleswig‐Holstein (UKSH) Campus Lübeck Lübeck Germany; ^6^ Department of Nephrology, Angiology and Rheumatology KRH Hospital Siloah Hannover Germany; ^7^ Institute of Medical Microbiology University Hospital Jena Jena Germany; ^8^ Department of Vascular Surgery Munich Hospital Bogenhausen Munich Germany; ^9^ Department of Dermatology, Venereology and Allergology University Medical Center Leipzig Leipzig Germany; ^10^ Department of Dermatology and Venereology University Hospital Halle Martin Luther University Halle‐Wittenberg Halle (Saale) Germany; ^11^ MSB Medical School Berlin University of Health and Medicine Berlin Germany

**Keywords:** definition, gram‐negative foot infection, infected interdigital intertrigo, recommendations, therapy

## Abstract

Infected interdigital intertrigo is an exudative, macerating, mixed infection of the toe webs in which gram‐negative bacteria (*Pseudomonas aeruginosa* and Enterobacterales) seem prevalent, but in which gram‐positive pathogens (*Staphylococcus [S.] aureus*, streptococci, enterococci) and fungi (dermatophytes, yeasts) also occur. The former German term “gram‐negative foot infection” misleadingly implied a soft‐tissue infection of the foot caused by gram‐negative bacteria. The updated term “infected interdigital intertrigo” is an internationally used designation, as is the term “gram‐negative toe web infection”. This superficial infection may yet progress to soft tissue infection (phlegmon, cellulitis) of the foot but in immunocompetent patients is usually caused by *S. aureus*. Complicated courses, as well as the development of interdigital intertrigo itself, are facilitated by diabetes mellitus, polyneuropathy, and peripheral arterial disease. Diagnosis is made clinically, based on painful macerated erosions or ulcers with yellow‐green exudate, putrid odor and edema. Superficial swabs are not always diagnostically required or relevant. Management focuses on local therapy with anti‐inflammatory, antiseptic, antimycotic agents, dry wound care and reduction of edema. Systemic antibiotics are indicated only in cases of associated soft‐tissue infection, which in immunocompetent patients present as uncomplicated cellulitis and therefore require coverage limited to *S. aureus*. Early vascular evaluation, imaging and surgical assessment in cases of deep infection or necrosis are essential.

## INTRODUCTION AND DEFINITION OF TERMS

While the term *gram‐negative foot infection* is traditionally used in the German clinical terminology, it is neither grammatically correct nor factually precise.

During pathogen diagnosis of this entity, different microorganisms are usually detected, and based on these findings alone it remains unclear which pathogen is causing and/or sustaining the infection. Apart from gram‐negative bacteria, gram‐positive bacteria and fungi are often isolated from the clinically relevant skin lesions (see chapter “Pathogen spectrum”).^1^ Infection of soft tissue arising from these lesions in immunocompetent patients is usually not caused by the gram‐negative pathogens isolated from intertrigo, but primarily by *Staphylococcus (S.) aureus*. Accordingly, antibiotics effective against staphylococci are sufficient for treatment.^1^, ^2^ The term “gram‐negative” is, therefore, misleading. Moreover, the word “infection” suggests a short, self‐limiting infection, while “foot infection” suggests an infection of the entire foot. There is, however, a localized infection originating from erosions or ulcers starting in the toe webs that will often spread to the dorsum of the foot in the form of diffuse erosions or ulcers. With the currently used term, the clinical differentiation from, for example, erysipelas or cellulitis, seems unclear.

Therefore, the guideline group suggests abandoning and replacing the term “gram‐negative foot infection”. A term based on the pathogenesis of the disease, already used in similar form internationally, should be adopted. Typically, the toe webs and the anterior aspect of the foot are infected. This is caused by a barrier defect – often in the form of a usually shallow ulcer originating from erosions in the toe webs in the form of intertrigo. It is, therefore, suggested that the term infected intertrigo of the toe webs, in short, infected interdigital intertrigo, be used given that this more clearly describes the clinical appearance and is not therapeutically misleading. In the Anglo‐American area, the term *toe web infection/intertrigo* is also known, although the attribute *gram‐negative* is still added occasionally, for example, *gram‐negative bacterial toe web infection* (GNTWI) or *gram‐negative bacterial toe‐web intertrigo*.^3^, ^4^, ^5^

**FIGURE 1:**
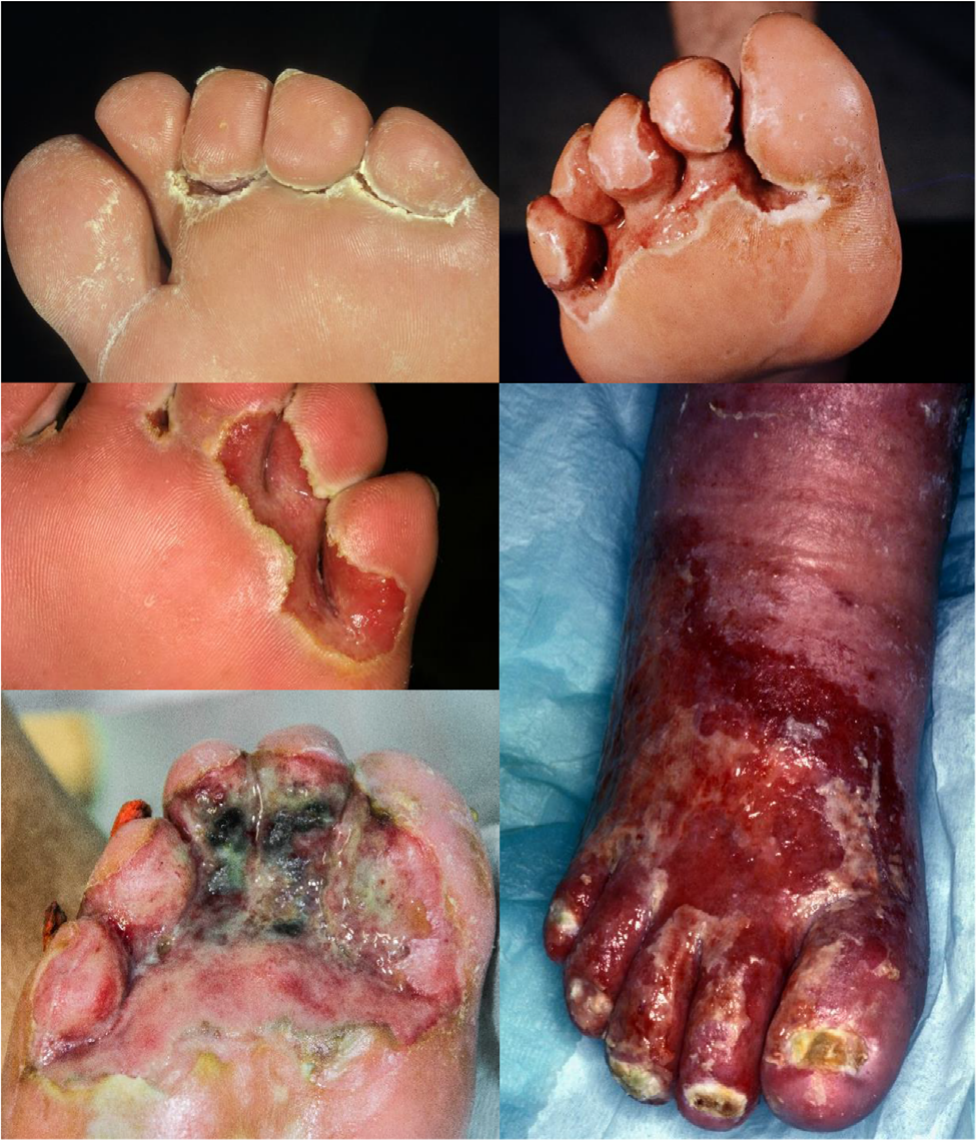
Clinical photos of infected interdigital intertrigo (Wound Center, Department of Dermatology, University Hospital Essen).

Typical clinical features are, in particular, the characteristic odor (sweetish/putrid) caused by gram‐negative colonization and the color caused by wet maceration (red exudative, whitish macerated) (Figure 1). In addition, edemas and pain are usually present.

Comorbidities, such as diabetes mellitus, polyneuropathy, or peripheral arterial disease (PAD), are common and promote the local infection.^6^ The barrier defect of the skin is caused by macerating interdigital tinea pedis or trauma.

**Table:** 

Definition: Infected interdigital intertrigo / so‐called “gram‐negative foot infection”
Exudative infected intertrigo of the toe webs beginning with macerations and erosions that may later spread to the anterior aspect of the foot forming ulcers. Typically, the dermatological disease is associated with the identification of various gram‐positive (staphylococci, enterococci, streptococci, and others) and gram‐negative (*Pseudomonas aeruginosa, Enterobacterales*, and others) pathogens as well as fungi. Often, comorbidities exist promoting the development of infected interdigital intertrigo. Infected interdigital intertrigo may progress to soft tissue infection (uncomplicated or complicated cellulitis), which, in immunocompetent patients, is caused by gram‐positive bacteria, such as *S. aureus*, rather than gram‐negative bacteria.

### Pathogen spectrum

The foot is subjected to particular stress and shows a rich colonization with various, to some extent changing, microorganisms for several reasons. The physiological colonization of the skin of the feet with microorganisms (microbiota) is extremely complex. It is presumed that there is a dynamic interaction between microbiota and host as well as within the microbiota.^7^

Factors that may lead to differences in the microbiome include:

− rather dry (for example, dorsum of the foot) and rather moist regions (for example, toe webs),
− feet are in contact with surfaces differing with respect to their bacterial colonization,
− changes in the microclimate by wearing socks and shoes with different ventilation resulting in differences in local temperature and humidity,^8^
− type, duration and localization of acute and chronic skin diseases and skin defects (including superficial to deep wounds),
− comorbidities of the host including local and/or systemic immune defects and circulation disorders as well as interventions with antibiotic/biocidal effect.^7^, ^9^ In this context, the microbial detection from diabetic foot ulcer (DFU) has been best studied.^9^, ^10^, ^11^

The results of microbiota analyses from skin surfaces are highly dependent on type and quality of the method used (molecular and/or culture‐based; use of both procedures is optimal). This includes sampling (swab or tissue) and taxonomic depth of the analyses.^10^, ^12^ They correspond only to a limited degree to the experience based on the analysis of skin swabs or other test materials from the foot in microbiological routine labs, which is often restricted to the identification of microorganisms with relevance for etiopathology. In accordance with the general knowledge about the colonization of human skin, studies of healthy volunteers or control groups show a typical colonization of the foot skin with representatives from four bacterial phyla. Most common are bacteria of *Staphylococcaceae* and *Streptococcaceae* as well as *Pseudomonadota* (synonymous *Proteobacteria*; including *Enterobacteriaceae*, *Moraxellaceae*, *Neisseriaceae*, and *Pasteurellaceae*), *Actinomycetota* (synonymous *Actinobacteria*; including *Corynebacteriaceae*, *Kytococcaceae, Micrococcaceae*, and *Propionibacteriaceae*), and *Bacillota* (synonymous *Firmicutes*; including *Bacillaceae*, *Clostridiaceae*, *Enterococcaceae*, and *Lactobacillaceae*); less often, species of *Bacteroidota* (synonymous *Bacteroidetes*) are found.^9^, ^13^, ^14^, ^15^ Overall, coagulase‐negative staphylococcus species appear to be dominating on the foot. This applies also to cultures from toe webs (identification in approximately 75 %), followed by corynebacteria (approximately 45 %), and micrococci (approximately 35 %); gram‐negative species are found in approximately 10 %.^15^, ^16^ Typical eukaryotic representatives on the foot skin are *Malassezia* species, followed by *Candida* species from the fungus “*kingdom*” and *Demodex* mites from the phylum *Arthropoda*. Moreover, fungi are often involved in skin infections in people with diabetes (prevalence of 84.6 % in type 1 diabetes). Apart from *Candida albicans*, this includes dermatophytoses of toe webs and nails.^17^

The *infected* interdigital intertrigo (IZI) presents mostly (22 %–90 %) as mixed infection.^4^, ^18^, ^19^ In culture‐based clinical studies, several different bacteria were isolated from erosions or ulcers in most cases (22 %–90 %).^11^, ^12^, ^13^ Typically, *S. aureus* and/or coagulase‐negative staphylococci (approximately 8 %–41 %), *Streptococcus pyogenes* (approximately 2 %–5 %), or *Enterococcus faecalis* (approximately 40 %) were among the gram‐positive‐microorganisms found in these culture‐based clinical studies ^2^, ^18^, while the most common isolates of gram‐negative bacteria included *Pseudomonas aeruginosa* (16 %–86 %), followed by *Enterobacterales* representatives (especially *Escherichia coli, Proteus mirabilis, Morganella morganii, Enterobacter cloacae, Klebsiella pneumoniae*, and *Serratia marcescens*) and *Acinetobacter species* with a prevalence of approximately 3 %–40 %.^5^, ^6^, ^18^, ^19^ In more than 50 % of the cases, fungal structures were detected by microscopy. If cultivable, these present predominantly as *Trichophyton (T.) rubrum* or *T. mentagrophytes* and as *Candida albicans*.^3^, ^5^

Accordingly, the results of microbiome studies and culture‐based analyses often revealed the presence of gram‐positive bacteria, such as *S. aureus* or *Enterococcus faecalis*, and not exclusively gram‐negative bacteria. Therefore, the intention expressed in the attribute “gram‐negative” foot infection appears to be unjustified.

### Differential diagnoses and complications

Table 1 provides an overview of the relevant differential diagnoses and potential complications. The key differences in clinical presentation are listed. There is no claim to completeness.

**TABLE 1:** Differential diagnoses and complications of infected interdigital intertrigo.

Ecthymas
− sharply defined, usually multiple, ulcerating infections of the skin caused by *S. aureus* or *S. pyogenes*, occurring usually on the legs and occasionally on the dorsum of the foot (often bilaterally) − in adults, there are usually either acute favoring conditions in the form of occlusion in warm and humid climate or poor hygienic conditions − initially, follicle‐bound pustules, from which a grayish‐yellowish crust develops − after detachment of the crust, shallow ulcer with sharply defined borders and surrounding erythema − spreading within a few days to 1–3 cm, then stable size
**Soft tissue infections of the foot**
***Erysipelas***
− pathogens are usually β‐hemolytic streptococci, most commonly of group A (*S. pyogenes*) and less often of groups B, C, and G − bright red, shiny erythema, sharply defined borders, often with arc‐shaped extensions, no suppuration − there is an entry port, for example, tinea pedis − elevated CRP levels and leukocytosis − general symptoms (shivering, chills, fever), usually at the onset of erythema formation − may be a consequence of infected interdigital intertrigo (“gram‐negative foot infection”), but considerably less common than cellulitis
***Uncomplicated cellulitis (phlegmon)***
− pathogen usually *S. aureus* − darker, more intensive livid erythema than in erysipelas − pasty swelling, different degrees of suppuration − entry port (ulcer) usually deeper than in erysipelas − not always associated with general symptoms (chills, fever) and if so, then not always initially, but also later − at onset, often no significantly elevated serological parameters, for example, CRP levels or leukocytes − may be the consequence of infected interdigital intertrigo − without complicating co‐factors, for example, PAD, diabetes mellitus with associated sequelae or insufficient metabolic control (≠ complicated cellulitis)
***Complicated or severe cellulitis (complicated soft tissue infection)***
− pathogen usually *S. aureus*, alternatively, infection or mixed infections with other gram‐positive and gram‐negative bacteria and anaerobes − soft tissue infection reaching to muscle fascia or muscle − associated with severe underlying diseases or other factors impeding response to therapy (the criteria for *complicated soft tissue infection* defined by the FDA for clinical trials apply^20^) − aggravating underlying diseases or factors include: insufficiently controlled diabetes mellitus, bacteremia, neutropenia (granulocyte count < 500/mm^3^), liver cirrhosis (Child classification B or C), chronic alcohol abuse, malnutrition, or immunosuppressive therapy − in presence of the above‐mentioned risk factors, it may be the consequence of infected interdigital intertrigo
**Foot infection in patients with insufficiently controlled diabetes mellitus (as a form of severe or complicated soft tissue infection (cellulitis))**: − pathogen spectrum includes relatively often gram‐negative bacteria and anaerobes; moreover, multiresistant pathogens are often causal agents already − renal insufficiency and other comorbidities interfering with the immune response − rapid spreading to deep tissue layers, adjacent tendons and within sheaths, joint capsules, bones (osteomyelitis), and into the bloodstream due to metabolic, neuropathic, and angiopathic damage caused by chronically increased blood glucose levels: ○ reduced immune response (among other factors, impaired function of neutrophils) ○ ischemia due to arterial occlusions (capillary system through to large peripheral vessels) ○ chronic DFU on mechanically stressed areas, for example, the toe webs, detected at a late stage due to diabetic neuropathy ○ hyperglycemic wound environment promoting the pathogenicity of *S. aureus‐*
***Necrotizing skin and soft tissue infection type I***
− rare aggressive infections resulting in their course in extensive, ischemia‐related destruction of tissue from epidermis to deep muscles due to the pathogenicity and virulence factors (especially toxins and enzymes) of certain (strains of) bacteria and their extensive proliferation due to the frequently compromised immune response^21^, ^22^ − toxin‐related shock symptoms and immediate threat to life − principal and only typical early symptom: disproportionate pain, that is, stronger than anticipated based on the clinical findings, caused by ischemia and necroses; crescendo‐like, only responding to morphine
***Gangrene***
− necroses on toes, edemas, cold skin with erythema of the distal foot, in case of pronounced edema and fluid in the skin of the toe web also wet gangrene − septic gangrene = occlusion of the supplying artery due to infection (clinical picture of blue “*sausage toe*”) and ischemic, typically dry gangrene susceptible to secondary infection

The specified (differential) diagnoses may become consequence or complication of infected interdigital intertrigo. Especially foot infections in patients with insufficiently controlled diabetes mellitus, either as DFU or toe‐web intertrigo, often progress to a complicated soft tissue infection and require particular attention, given that they may rapidly spread to deeper tissue levels, bones (osteomyelitis), and into the bloodstream.

### Diagnostic workup

#### Initial clinical diagnosis

**Table:** 

**Recommendation**
**Clinical** The initial diagnosis is made based on the following **clinical** findings:
− diffuse erosions, later potentially also ulcerations that may spread from the toe webs to the dorsum of the foot − yellow exudate, yellow‐greenish coatings, potentially pus − maceration at the border of the barrier defect − sweetish‐putrid odor − edema − pain − absent tendency to heal; often, gradual increase in size (pus or absence of healing, increase in size, pain, and increasing redness of the wound edge are the criteria for local infection of ulcers) − often, tinea pedis with interdigital maceration or other previous traumas − history of typical comorbidities promoting the local infection, such as chronic lymphedema, diabetes mellitus, polyneuropathy, or PAD

### Pathogen diagnosis

**Table:** 

**Recommendation**
To confirm the clinical diagnosis, **pathogen identification including resistance testing may** **be performed**. In complicated or necrotizing processes, we **recommend pathogen identification including resistance testing**.
− When interpreting a superficial wound swab, it must be taken into account that this is of little informative value, given that colonizing and secondarily infecting bacteria are also collected. − If a wound swab is performed, it should be taken from the infected wound edges, the wound bed, or tissue of wound crusts. − The microbiological identification of gram‐negative rods (most commonly, *Pseudomonas aeruginosa* and *Enterobacterales*) in addition to identification of *S. aureus* or β‐hemolytic streptococci is typical in infected interdigital intertrigo, but has no further clinical relevance. − If indicated and obtainable, and especially if signs of complicated (severe) cellulitis and necrotizing infections are observed, native tissue samples (biopsies) from wound edges or wound bed should be preferred over swabs, given that they will allow for the most sensitive and specific identification of pathogens of soft tissue infections by cultivation or DNA‐based methods; cultivation succeeds best prior to administration of antibiotics.

Vital pathogens causative for the infection are localized at wound edges, wound bed, or under the wound crusts. A swab should be taken from various localizations, if possible (without touching non‐lesional areas of the wound edge). Superficial swabs from erosions or ulcers are, therefore, of little informative value and result in inappropriate antimicrobial therapy due to misinterpretation.^23^ However, if a swab is taken, so‐called “flocked” nylon swabs with Amies transport medium should be used. Before taking the swab, superficial secretions and fibrinous or necrotic coatings must be removed in a sterile manner.

If indicated and obtainable, *native* tissue samples should be preferred for reasons of sensitivity, given that they allow for both cultivation of presumably relevant bacteria and use of nucleic acid‐based identification methods, either in parallel or after negative cultures. However, collecting tissue samples is mainly useful if clinical signs for spreading of the infection into soft tissue (dermis, subcutis, or deeper layers) are observed (also for cellulitis) and if antibiotics have not yet been administered (since experience shows that even cultivation of bacteria resistant in vitro will fail). It is predominantly indicated in cases with severe or fast‐spreading complicated cellulitis. For this purpose, it is recommended to divide the tissue sample and store the aliquots temporarily (depending on the duration at 5 °C to ≤ –20 °C). If histopathological examinations are required in parallel, the tissue material should be split for pathology and microbiology (not fixed in formalin). If only formalin‐fixed/paraffin‐embedded tissue samples are available, molecular methods may be used in the form of a *rescue* procedure, although with significantly lower sensitivity and higher risk of contamination.^24^ For ulcerations, excisions obtained under sterile conditions are most appropriate.^23^ The tissue samples should be taken from the reddened, infected area, approximately 1 cm from the wound edge, after prior thorough antiseptic cleansing of the skin surface to avoid contamination of the sample with pathogens from the ulcer. It is the goal to determine which bacteria below the intact epidermis are responsible for soft tissue infection.^23^ In view of the inhomogeneous distribution of pathogens in infected tissue, a relatively high pathogen density is anticipated near the wound edge.

Sterile transport containers must be used for tissue samples. Dehydration of the tissue samples must be prevented by adding sterile physiological saline. Given that transport and storage times reduce the diagnostic yield, transport times must be kept short, ideally below two hours. If a transport time of 2–4 hours is exceeded, refrigerated transport of samples is generally recommended.

For cultivation, a broad spectrum of pathogens should be considered, including anaerobes (see *Mikrobiologisch‐infektiologische Qualitätsstandards (MiQ) “Infektionen der Haut und der subkutanen Weichgewebe”* [Microbiological‐infectiological quality standards, infections of skin and subcutaneous soft tissues]^23^). Inoculated agar plates are cultivated for 48 hours; a first reading is performed after 24 hours. Broth tubes are controlled for growth daily for 7 days.

For the detection of rare pathogens and diagnostic strategies for screening or detection of multi‐resistant pathogens (MRPs), we refer to the literature.^23^ When interpreting the microbiological findings it is important to consider that *(1)* it is generally difficult to differentiate between colonization and infection, in case of inappropriate sampling also contamination, if samples are obtained from primarily non‐sterile areas, *(2)* pathogens are distributed inhomogeneously in infected tissue, and *(3)* vitality and, therefore, ability of cultivating bacteria is reduced depending on the strength of the immune response or after prior treatment with antibiotics (even in case of potential resistance).

### Assessment of severity

There are currently no validated staging tools for the assessment or grading of the severity of infected interdigital intertrigo.

Localization, manifestation, and extent of macerations, erythema, edemas and erosions, as well as ulcerations should be described. Photographic documentation facilitates the evaluation of the disease course.^25^ For better comparability and prediction, the wound conditions should be described in a structured manner. This is facilitated by using one of the common wound classifications.

The local infection in the form of infected interdigital intertrigo should be differentiated from soft tissue infection in the form of cellulitis – which may originate from it.

The internationally accepted PEDIS classification of the *International Working Group on the Diabetic Foot* (IWGDF) is suitable for describing the extent (for example, in the framework of studies).^26^ However, this classification applies primarily to foot infections in diabetes mellitus and describes, therefore, parameters relevant in this context: perfusion, extension, and depth of the wound, existing local signs of inflammation, and disturbed sensation. It is also used in the German guideline on treatment of skin and soft tissue infections.^2^

In the most recent update, the IWDGF favors the use of the SINBAD classification system and score. This is an acronym consisting of six elements graded according to their severity: site of ulceration, ischemia, neuropathy, bacterial infection, area, and depth. The total score ranges between 0 and 6 and is divided into three categories referring to the risk of amputation of the lower limbs.^27^ If the clinical (ischemic rest pain, cool skin, prolonged capillary refill time) and instrument‐based diagnostic workup provided evidence for the presence of PAD, the benefit of vascular intervention and the risk of a potential amputation may be assessed by the WIFI system comprising the criteria wound, ischemia and foot infection (WIFI). It is based on the classification of the *Infectious Disease Society of America* (IDSA)/*International Working Group on the Diabetic Foot* (IWGDF) for assessment of infection. Amputation rates and healing duration of the wound correlate with WIFI. The WIFI system is primarily used for scientific purposes. For the assessment of patients with diabetes, however, the system lacks the criterion “neuropathy”.^28^, ^29^

For assessment of foot infection in people with diabetes mellitus, the IWGDF currently recommends:

− the classification SINBAD for clinical use and the comparison of results (audit),
− the WIFI system for detecting infection and perfusion and assessing the benefit of revascularization.^27^, ^30^, ^31^

The most severe form of soft tissue infections are the so‐called necrotizing soft tissue infections presenting an immediate vital threat; the provisional diagnosis is made clinically (see chapter “Differential diagnoses”). The *Laboratory Risk Indicator for Necrotizing Fasciitis (LRINEC) Score* may assist in differentiation from complicated, non‐necrotizing cellulitis.^32^, ^33^, ^34^, ^35^

No classification system is able to reliably predict the prognosis and course of either infection or ulcer.

### Advanced diagnostic measures

**Table:** 

**Recommendation – diagnosis of spreading**
Regular clinical control to detect evidence of soft tissue infection (usually cellulitis) in time, for example, spreading erythema or swelling. Apart from the clinical findings, CRP levels and leukocytes may provide evidence of cellulitis and allow assessment of the therapeutic response (taking the typical latency into account, for CRP 48–72 hours). Given that in foot infections of patients with insufficiently controlled diabetes mellitus, cellulitis may spread rapidly into the depth, diagnosis of spreading by respective clinical and imaging methods should be performed at an early stage.^2^, ^36^, ^37^ If a deeper infection with involvement of tendons, joint capsules, or bones is suspected or in case of severe pain, additional diagnosis of spreading with imaging techniques. Prior to antibiotic treatment, factors suspicious of osteomyelitis should be assessed.^38^ Factors suspicious of osteomyelitis include:^39^ ○ ulcers not healing properly despite adequate wound treatment and absence of relevant ischemia or PAD, ○ ulcers with a size of more than 2 cm^2^ and a depth of 3 mm,^38^ ○ bone contact in exploration, ○ exposed portions of bone, ○ bacterial dactylitis (sausage‐like swollen toes), ○ pathological ESR (> 70 mm), elevated CRP levels, elevated procalcitonin levels, leukocytosis.

**Table:** 

**Recommendation – imaging**
In case of symptoms corresponding to a clinically suspected spread of the infection, we **recommend** imaging of the respective foot.
− Sonography may be sufficient for circumscribed abscesses. − For deeper infections of unclear extension and strong suspicion of osteomyelitis, diagnosis with tomographic techniques is appropriate to diagnose the total extension of an abscess‐ or cellulitis‐forming process with potential osteomyelitis. − MRI provides the highest informative value. If contraindications for MRI exist or MRI is not available, CT is an alternative option.^40^ If MRI or CT examinations are not possible or available, at least x‐ray imaging of the foot skeleton in two planes should be performed in the short term to exclude osteomyelitis. − If osteomyelitis is still suspected or if tomography did not achieve clarification, ^18^F‐FDG‐PET or ^99^mTc‐HMPAO‐labeled leukocyte scintigraphy may be performed. − If osteomyelitis is suspected, this should be confirmed by taking a bone biopsy for microbiological and histological examination (in case of prior administration of antibiotics not before 15 days after discontinuation of the antibiotic).^38^

**Table:** 

**Recommendation – vascular diagnosis**
For the clinical examination, we **recommend** including a diagnostic workup of the arterial and venous supply of the affected extremity (see below) including imaging if a corresponding disorder is suspected. We **recommend** angiological examination or counseling with vascular surgery in case of reduced ankle‐brachial index, any other clinical suspicion of vascular pathology, or absent improvement of findings on adequate therapy without apparent reason. We refer to the S3 guideline “Diagnostics, treatment and aftercare of peripheral arterial occlusive disease” (AWMF registry number: 065‐003) and the joint position paper of the German societies for diabetes, angiology, interventional radiology, and vascular surgery.^41^ Individual measures are listed here:
**Arterial**
− To assess the presence of PAD, the ankle‐brachial index (ABI) should be determined (systolic Doppler occlusion pressure measurement in rest on both upper arms and distal lower legs, normal 0.9–1.3). − In case of ABI values > 1.3 with suspected medial calcific sclerosis (in approximately 30 % of people with type 2 diabetes, erroneously elevated value due to impaired compressibility), otherwise implausible occlusion pressures, or other indications for PAD (cold toes, iris diaphragm phenomenon, history‐related evidence), supplemental methods, such as toe‐brachial index (TBI), analysis of the pedal Doppler frequency spectrum, or oscillography, should be used. − From ABI < 0.9, TBI < 0.7 or in case of a monophasic Doppler waveform, an advanced angiologic diagnostic workup should be performed. − In case of critical ischemia (ABI < 0.5, transcutaneous partial pressure of oxygen tcpO_2_ < 30 mmHg), there is acute need for action to prevent ischemic complications. − In the advanced diagnostic workup, color‐coded duplex sonography (CCD) of leg and pelvic arteries has a key role for assessing the need and planning of measures for revascularization. Based on its results, the decision should be made for performing angiography in readiness for intervention or additional imaging procedures, such as MR or CT angiography.
**Venous**
− CCD of pelvic and leg veins is indicated if deep vein thrombosis is suspected.

### Assessment of comorbidities

**Table:** 

**Recommendation**
In infected interdigital intertrigo, we **suggest** to look for clinical signs of the following diseases and to arrange for additional diagnostic and therapeutic steps, if necessary:
− diabetes mellitus − lymphedema − chronic venous insufficiency − PAD

Studies have shown that foot infections occur frequently in people with diabetes mellitus. For example, in the largest Israeli study collective on infected interdigital intertrigo with 200 affected patients, a proportion of 16 % with diabetes mellitus was described. Notably, these patients had significantly elevated CRP levels compared to patients without diabetes mellitus. This indicates that people with diabetes have a tendency of developing more severe disease courses. Other comorbidities, such as arterial hypertension or dyslipidemia, seem to have no effect on disease severity.^6^ Due to hyperglycemia‐induced metabolic and immunological changes, such as reduced T cell reaction and neutrophil function, and due to PAD or occlusive arteriolopathy, people with diabetes mellitus are more susceptible to skin infections. The hyperglycemic wound environment promotes the pathogenicity of *S. aureus*.^42^ The frequently present diabetic neuropathy results in decreased perception of pain and thus in an unnoticed progress of the infection that will, moreover, spread rapidly (via tendons) from compartments with high to compartments with lower tissue pressures (see above). The clinical diagnostic workup should include examination of a potential pathological pressure load on the foot skin due to neuropathic deformity of the foot and sensitivity tests or assessment for the presence of sensory neuropathy by tuning fork, TipTherm^®^, and monofilament. The risk of severe infections is enhanced in case of previous foot lesions or amputations.

### Therapy

#### Local therapy

**Table:** 

**Recommendation**
We **recommend** performing the local therapy irrespective of the severity of toe‐web intertrigo. We **suggest** pursuing the following primary therapeutic goals:
reduction of inflammation reduction of bacteria and fungi, if applicable reduction of “wet chambers” reduction of edema

**Ad 1)** At the start of treatment, local therapies with wraps containing, for example, synthetic tannins, such as Tamol, are recommended in combination with antiseptics (see ad 2). These tannins have an anti‐inflammatory and astringent effect.^43^ Short‐term topical use of ultrapotent glucocorticoids is useful in case of strong inflammatory activity.^44^ During selection of the used formulation, the principle “wet on wet, dry on dry” should be observed (for exceptions, see ad 3). Accordingly, alcohol‐free lotions are primarily used for acute treatment.

**Ad 2)** Bacteria and wound coatings may partially be removed already by mechanical debridement, for example, with sterile cotton compresses.^45^, ^46^ Special cleansing pads or sponges present an alternative option.^47^ To further reduce the number of bacteria and fungi, modern antiseptics with low cytotoxicity containing polyhexanide (PHMB) or octenidine (with phenoxyethanol), potentially also with povidone (PVP) iodine, are used, for example, by application with wet wraps.^48^ The respective exposure times of these antiseptics (PHMB 10–20 minutes, octenidine 1–2 minutes, PVP iodine 3–5 minutes) have to be observed.^49^ The addition of agents like potassium permanganate, dyes, hydrogen peroxide, or quinolinol is not recommended by the guideline group, given that they are more cytotoxic, more prone to errors during preparation or dilution, and because drug quality and quantity of the dyes cannot be assured. Moreover, topical antibiotics, such as gentamycin, as well as silver sulfadiazine should no longer be used, given that they act insufficiently on bacteria resistant to antibiotics, may select for resistant colonizing bacteria, and may mediate immune activation with subsequent drug reaction.^48^ If tinea pedis was identified, topical antimycotic treatment should be performed not later than after termination of antiseptic treatment.

***Ad 3)*** In contrast to many other wounds, infections of the frequently wet toe webs (especially if the impact of gram‐negative bacteria is clinically evident based on odor, greenish wound exudate, and pus) are usually treated by desiccating treatment measures to restrict the local environment favoring humidity‐loving pathogens. In this context, the insertion of cotton compresses or other soft textiles in the toe webs is of particular importance to prevent the formation of so‐called wet chambers or of intertrigo and to transport moisture to the outside.^50^ For this purpose, medical textiles are available containing silver with antimicrobial activity.^51^ In addition, exudate‐binding secondary dressings up to superabsorbers should be used for heavily exuding wounds.^52^ However, the tissue should not become too dry, given that a moist wound environment is required for most processes involved in wound healing.

***Ad 4)*** In case of pronounced edemas, medical compression therapy should be performed after excluding contraindications, such as advanced peripheral arterial disease (ABI < 0.5; ankle systolic blood pressure < 60 mmHg, toe blood pressure < 30 mmHg, tcpO_2_ < 20 mmHg^53^), decompensated heart failure (NYHA III + IV), and phlegmasia cerulea dolens.^54^ Usually, a low application pressure of around 20 mmHg from the metatarsophalangeal joints to the knees at the start of compression is sufficient for effective edema reduction.^55^

Exemplary presentation of the practical process of local therapy of acute, inflammatory, gram‐negative foot infection:

1. wet wraps for approximately 10 minutes with additives, for example, tannins
2. mechanical cleansing, for example, with sterile cotton compresses,
3. application of wraps, for example, with octenidine and phenoxyethanol for at least two minutes
4. thin application of, for example, betamethasone lotion
5. insertion of sterile cotton compresses in toe webs, with secondary dressing, if appropriate
6. in pronounced edema, application of medical compression dressing with application pressure of at least 20 mmHg

Intervals of dressing change are based on the respective amount of exudate. At treatment start, changing several times a day may be necessary. Later in the disease, items 1, 4, and 6 are often dispensable; daily dressing changes are then no longer required.

In addition, iontophoresis may be considered in case of concomitant plantar hyperhidrosis in the long term.^56^

### Systemic therapy

**Table:** 

**Recommendation**
In cases with **concomitant soft tissue infection** (usually uncomplicated or complicated cellulitis [see Chapter “Differential Diagnoses”]), we **recommend** systemic antibiotic therapy according to the S2k guideline “Calculated parenteral initial therapy of bacterial infections in adults – AWMF registry number 082‐006”*. Accordingly, the antibiotic therapy should be effective against *S. aureus*, in particular (cefazolin or flucloxacillin parenteral). In general, treatment duration of 5 days is recommended.^57^, ^58^ However, the treatment duration should also be guided by the clinical response. *The S2k guideline was written in 2017 and is currently under revision. Systemic antibiotic therapy is not generally indicated in infected interdigital intertrigo, given that it is primarily a superficially infected erosion or ulceration. Therefore, detection of pathogens from wound swabs by culture without clinical signs of soft tissue infection does not justify a systemic antibiotic therapy. In cases with rapidly progressing cellulitis or if cellulitis is unresponsive to the therapy mentioned above, aminopenicillin plus beta‐lactamase inhibitor (amoxicillin/clavulanic acid or ampicillin/sulbactam) is recommended due to the potential presence of gram‐negative bacteria in infected intertrigo (and the frequently present diabetes mellitus).
During treatment, we *suggest* reevaluating and, if necessary, adjusting the antibiotic therapy once the results of antibiotic susceptibility testing from tissue samples are available (see Chapter “Pathogen diagnosis”).

In the past, fluoroquinolones were regularly used for systemic therapy of gram‐negative foot infection or toe‐web intertrigo. However, these are no longer recommended because of the usually lacking indication^2^ and the narrow therapeutic range. Moreover, there is an increased risk of impairing and irreversible musculoskeletal and neurological side effects (Commission Implementing Decision (EU) C(2019)2050 and Risk Assessment Procedure pursuant to Article 31 of Directive 2001/83/EC).

For the reasons mentioned above, the bioavailability of oral antibiotics is not adequately assured. Local therapy with antibiotics must be rejected, given that it is not sufficiently effective, selects for resistant bacteria, and may cause sensitization to the respective group of antibiotics via the immune system of the skin. For the specified antiseptics, however, the topical efficacy is better assured and resistance development is less likely.

Although a literature search on soft tissue infections in infected interdigital intertrigo was performed, it was not productive and the corresponding results were not always easy to compare due to the narrow formulation of the question, the frequently unclear use of terms (local infections, complicated or uncomplicated cellulitis), and due to the different microbiological examinations (swabs vs. tissue samples).^4^ As an example, excerpts of an oriented literature search performed during guideline development are mentioned here. This illustrates the heterogeneity of care and the, in our view, unnecessary use of antibiotics with broad spectrum and high risk of adverse drug effects:

In a retrospective multicenter French study on *gram‐negative toe web infection*, 45.2 % of the patients (overall, n = 62) received systemic antibiotic treatment in addition to local therapy. In 57 %, it was directed against *Pseudomonas aeruginosa* and resulted in administration of piperacillin/tazobactam with or without ciprofloxacin.^44^ Others, however, received antibiotics specific for *S. aureus* or β‐hemolytic streptococci, including the patients with *cellulitis* (not further defined) (24 %), with as much treatment success.^44^ In a prospective monocentric study, 123 patients were treated systemically with ciprofloxacin or intramuscular injection of ceftazidime or cefotaxime, but the number of successfully treated patients was not stratified by the individual therapeutics.^5^

For the selection of antibiotic systemic therapies, the guideline group refers to the S2k guideline *“Calculated parenteral initial therapy of bacterial infections in adults” AWMF registry number 082‐006*,^59^ in particular to Chapter 9 (starting on page 173) on skin and soft tissue infections (an update is in progress).^2^ Accordingly, antibiotic therapy against *S. aureus* would be indicated initially (reported from page 183 onward).^2^, ^39^ This approach has proven successful in daily clinical practice of the authors of the present guideline.

### Surgical options

**Table:** 

**Recommendation**
We **recommend** focusing on multimodal local therapy (see Chapter “Local therapy”) for treatment of toe‐web intertrigo and on administration of antibiotics in case of soft tissue infection (see Chapter “Systemic therapy”). We **recommend** surgical evaluation in the following concomitant conditions or provisional diagnoses:
− deep infections (involvement of deeper soft tissue, for example, fascias and muscle layers, analogous to the definition of complicated skin and soft tissue infections^2^, ^60^, ^61^ − osteomyelitis − severe circulation disorders of the extremities − clinical signs of compartment syndrome − signs of necrotizing soft tissue infection (see Chapter “Differential diagnoses”)

The surgical therapy comprises surgical debridement of inflamed and, if applicable, already necrotic skin or, if necessary, even amputation of toes or the entire digital ray in ischemic gangrene or deep and systemic infection.^22^, ^62^

Due to the foot anatomy with long muscle and tendon compartments, infections may easily spread proximally. The inflammatory reaction – caused by the infection – may result in an increase of the compartmental pressure that may aggravate existing tissue ischemia or cause such an ischemia. While classical compartment syndrome in young men (< 30 years of age) is more likely to occur after fractures or traumas, variants have also been observed in unfavorable constellations or if co‐factors exist (alcohol abuse, infection).^63^ Classical symptoms include: rapidly increasing, disproportional pain of one limb not in line with the external finding, rough to firm muscle on palpation, pain upon stretching the fascial compartment, sensory and motor neurologic deficits. In case of sudden pain uncontrollable by NSAIDs (*non‐steroidal anti‐inflammatory drugs*), necrotizing soft tissue infection and arterial embolism (presenting with pale tissue, pulselessness, and paresthesia) must be excluded in differential diagnosis.

While necrotic soft tissue infection (NSTI) is rare, diabetes mellitus or diabetic foot ulcer^22^, ^64^, ^65^ as well as decubitus ulcers, higher age, and immunosuppression are risk factors. Again, principal and only typical early symptom is disproportional, crescendo‐like pain caused primarily by the ischemia and responding only to morphine.^2^ Given this risk, prompt surgical assessment of soft tissue infection is essential, especially for limbs with impaired circulation.^22^ Standardized evaluation (for example, WIFI score) should be performed to assess the extent of soft tissue infection (see also Chapter “Assessment of severity”).^22^, ^25^, ^28^, ^29^, ^60^, ^62^, ^66^, ^67^

### Prevention – treatment of tinea pedis

**Table:** 

**Recommendation**
− In case of existing tinea pedis and/or onychomycosis, we **recommend** antimycotic therapy for prevention of toe‐web intertrigo.

Interdigital tinea pedis is considered a predisposing factor for macerations and secondary colonization by gram‐negative bacteria (damage of the skin barrier, displacement of microbiota by production of antibacterial substances with promotion of colonization by gram‐negative bacteria).^4^ Moreover, in a case series, all 15 patients with so‐called gram‐negative foot infection reported a history of recurrent tinea pedis.^6^ The most common pathogens are *Trichophyton (T.) rubrum* and *T. interdigitale*, while yeasts are only occasionally detected and molds usually not at all.^23^, ^68^ Treatment is primarily topical, except for tinea pedis of the dyshidrosiform type and the “moccasin” type, which are additionally treated by oral administration of terbinafine for 14 days.^69^, ^70^ In principle, all available substance classes of topical antimycotic agents may be used. The allylamine terbinafine has the advantage that it is generally used only once daily for a short treatment duration of 7 days. While terbinafine has no additional antibiotic effect against gram‐negative bacteria,^71^ this has been demonstrated for ciclopirox,^72^, ^73^, ^74^ which presents, therefore, a therapeutic option. It is applied in the morning and evening until 1–2 weeks after resolution of the inflammation, usually for 3–4 weeks.^75^ Initially, shoes and stockings or socks should be treated accordingly with antimycotic spray and antimycotic detergent. Preventive treatment measures include intensive drying, especially of the toe webs, after bathing or showering, wearing of clean, non‐occluding socks and shoes, preferably made of cotton or other natural fibers, and treatment of associated plantar hyperhidrosis.^4^, ^69^, ^76^

From a preventive point of view, treatment of existing onychomycosis is recommended. In addition, we refer to the S1 guideline on onychomycosis (AWMF registry number: 013‐003, 2022).^77^

For the sections “Differentiation from other guidelines/disciplines”, “Limitations of the guideline”, “Information about this guideline” (including “Handling of conflicts of interest” and “Funding”), and “Methods” (including “Generation of recommendations”, “Approval of the guideline”, and “Exploitation rights”), see long version.

## CONFLICT OF INTEREST STATEMENT

A complete list of the specified conflicts of interest is available at: https://register.awmf.org/assets/guidelines/013‐109l_S1_Infizierte‐Zehenzwischenraum‐Intertrigo‐gramnegativer‐Fu%C3%9Finfekt__2025‐09.pdf
